# A database resource and online analysis tools for coronaviruses on a historical and global scale

**DOI:** 10.1093/database/baaa070

**Published:** 2020-10-03

**Authors:** Zhenglin Zhu, Kaiwen Meng, Gexin Liu, Geng Meng

**Affiliations:** School of Life Sciences, Chongqing University, No. 55 Daxuecheng South Rd., Shapingba, Chongqing, 401331, China; College of Veterinary Medicine, China Agricultural University, HaiDian District, Beijing, 100094, China; School of Life Sciences, Chongqing University, No. 55 Daxuecheng South Rd., Shapingba, Chongqing, 401331, China; College of Veterinary Medicine, China Agricultural University, HaiDian District, Beijing, 100094, China

**Keywords:** coronavirus, SARS-CoV-2, 2019-nCoV, COVID-19

## Abstract

The recent outbreak of COVID-19 caused by a new zoonotic origin coronavirus (SARS-CoV-2 or 2019-nCoV) has sound the alarm for the potential spread of epidemic coronavirus crossing species. With the urgent needs to assist disease control and to provide invaluable scientific information, we developed the coronavirus database (CoVdb), an online genomic, proteomic and evolutionary analysis platform. CoVdb has brought together genomes of more than 5000 coronavirus strains, which were collected from 1941 to 2020, in more than 60 countries and in hosts belonging to more than 30 species, ranging from fish to human. CoVdb presents comprehensive genomic information, such as gene function, subcellular localization, topology and protein structure. To facilitate coronavirus research, CoVdb also provides flexible search approaches and online tools to view and analyze protein structure, to perform multiple alignments, to automatically build phylogenetic trees and to carry on evolutionary analyses. CoVdb can be accessed freely at http://covdb.popgenetics.net. Hopefully, it will accelerate the progress to develop medicines or vaccines to control the pandemic of COVID-19.

## Introduction

Coronaviridae is a group of positive-sense, single-strand RNA viruses with a likely ancient origin, and human coronavirus repeatedly emerged during the past hundred years (1). Coronaviruses are classified into four distinct genera: alpha and beta coronavirus mainly infect mammals, whereas gamma and delta coronavirus circulate more often in avian hosts (2). As a potential dangerous zoonotic disease, the previous outbreaks of respiratory syndrome-related coronavirus (SARS-CoV) and Middle East respiratory syndrome-related coronavirus (MERS-CoV) have plagued the general public and researchers in the past years (3). Recently, a novel coronavirus, which may originated from wild animals, was first identified in Wuhan City, China. The virus is the severe acute respiratory syndrome coronavirus 2 (SARS-CoV-2), also named 2019 novel coronavirus (2019-nCoV), causing coronavirus disease-2019 (COVID-19). Till now, it has resulted in more than 16 million confirmed infections worldwide (4, 5) with the number of infection cases still increasing. Although we have knowledge and experience in the virology, diagnosis, clinical characteristics and other aspects related to SARS-CoV and MERS-CoV, there are many unanswered questions about the new emerging SARS-CoV-2. The new outbreak coronavirus strongly reminds the continuous threat of zoonotic diseases caused by coronavirus to global health security. Sharing experience and knowledge across disciplines in historical and global scale should provide invaluable scientific knowledge to fight against the threat of coronavirus.

The aim of the construction of CoVdb is to provide coronavirus knowledge, to contribute to global coronavirus research, especially for the investigation of the emerging SARS-CoV-2. For previous works, ViPR (6) and ViralZone (7) are general data resources and are lack of analysis tools in population genomics and evolution. Different from those databases, CoVdb is specially designed for coronavirus. It combines, compares and annotates all published coronavirus genomes up to date (8–19). Compared to 2019nCoV (20), CoVdb provides more population genetic analysis information and contains several online sequence analysis tools. The new developed database provides the convenience for the identification of gene function and identity among Coronaviridae genomes. CoVdb provides information on subcellular location, function, protein topology, as well as population level through analyses. We will be dedicated to keep updating the genomic information and optimizing the database.

## Materials and methods

### Data processing

Coronavirus sequences and annotations are downloaded from the NCBI nucleotide database (21). We chose records with complete genomes. For newly sequenced strains without open reading frame (ORF) annotation, we did annotation through mapping known coronavirus proteins to the genome by GeneWise (22). We verified the quality of these proteins by known proteins, documented coronavirus proteins in NCBI and kept predicted proteins that have an identity >0.5 and a coverage >50%. We renamed all human isolates in the format of ‘Human_name_accession’ (‘name’ is 2019-nCoV (SARS-CoV-2), SARS, MERS or other human coronavirus strain names) and all nonhuman isolates in the format of ‘host_accession’ (‘host’ is bat, camel, cow or other coronavirus hosts). Accession is the strain’s GeneBank ID. According to previous methods to cluster homologous genes (23, 24), we grouped coronavirus proteins into 628 unified clusters by CD-HIT (25) with an identity >50% and a coverage >80%. We made classification for all documented coronavirus strains based on NCBI’s annotation on taxonomy. For an unclassified strain, we run BLAST with the strain’s genome against a ‘reference set’ of some lineage, such as Sarbecovirus, Setracovirus and so on. According to the BLAST output, we attribute the strain to a closest lineage. We list the sequenced strains documented in GISAID (26), and users are instructed to visit GISAID if they want to get the actual data.

We wrote Perl scripts to automatically BLAST coronavirus protein sequences against the UniProt database (27). We filtered out hits with an *E*-value <0.05 and only kept the one that has the highest score in alignments. Using matched UniProt accession numbers, we retrieved detailed proteomic information from UniProt. We looked for each gene’s possible protein 3D structure counterparts in the Protein Data Bank (28, 29) in the same way.

We did subcellular localization prediction for coronavirus gene clusters using an online tool MSLVP (30). We used TMHMM 2.0 (31) to predict the transmembrane helixes within protein sequences for all coronavirus genes and converted output images into PNG format by Magick (www.imagemagick.org).

We utilized CUDA ClustalW (32) to perform multiple alignments of all documented coronavirus genomes or proteins. The results are used to build phylogenetic trees. We aligned genomes by LASTZ (33), aligned CDS or protein sequences by MUSCLE (34, 35) and built phylogenetic trees by FastTree 2.1 (36). In the detection of selection signals, we performed sliding widow analyses with a window size 200 bp and a step size 50 bp. Population genetic tests were performed by VariScan 2.0 (37, 38) and SweepFineder2 (39). Due to the lack of software able to use multiple alignment to calculate the Fixation Index (Fst) (40), we wrote Perl scripts to calculate Fst according to reported algorithms (41).

A semi-automatic pipeline was developed to update the database, considering the fast increase of coronavirus’ sequences in NCBI. For specifics, the pipeline first refreshes the strain list according to NCBI’s coronavirus list (requiring complete genome), then updates corresponded annotation and finally make revision for some overall analysis results.

### The development of engines, interfaces and tools

Similar with what we have done for SGID (42) and ASFVdb (43), we made the web interface of CoVdb based on SWAV (44). CoVdb also incorporates MSAViewer (45) to display multiple alignments and phylotree.js (46) to show phylogenetic trees. To improve the display of virus data, we made changes in these two softwares. We changed parameters to fit virus’s dense gene arrangements and added links within diagrams. The search engine is written by PHP integrated with SQL, BLAT (47) and NCBI BLAST (48). The protein 3D viewer in CoVdb uses the libraries of iCn3D (49). For ‘Pop Analyzer’ and ‘Aln Browser’, the background codes were written basing on C, Perl, VariScan 2.0, SweepFineder2, FastTree 2.1 and LASTZ. ‘Phylo Tree’ shows phylogenetic trees built by genomes or coronavirus’ major proteins, the Orf1ab polyprotein (Orf1), the spike glycoprotein (S), the envelope protein (E), the membrane protein (M) and the nucleocapsid protein (N).

**Figure 1.**
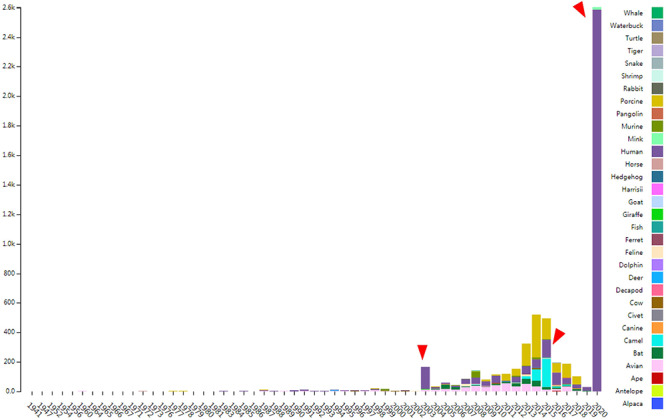
The distribution of documented coronavirus strains in CoVdb according to collection date (X-axis) and hosts (colored by different colors). Y is the number of coronavirus isolated from some organism. Red triangles points to peaks in the distribution of human coronavirus in years.

## Results and discussion

### Data and information

**Figure 2.**
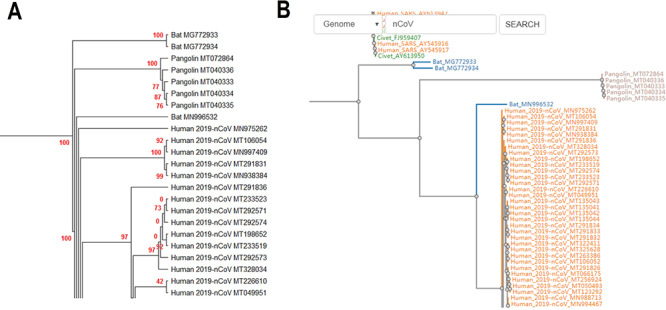
(A) Partial display of the phylogenetic tree built by all coronavirus genomes documented in CoVdb. Red numbers are marginal likelihoods. (B) Snapshot showing that users can search a strain by name in a phylogenetic tree. Both A and B center on the split of Bat_MN996532 and 2019-nCoV (SARS-CoV-2).

CoVdb extensively collects published coronavirus data and have taken in genomes of 5709 strains after the update in 22 May 2020. The strains were collected from 32 organisms and in the years from 1941 to present, 2020 (Figure 1). A total of 3414 (59.8%) in CoVdb are human isolates and 217 (3.8%) are bat isolates, which are referred as the possible source of human coronavirus (50, 51). Porcine coronavirus also take a big percentage (945, 16.6%) and coronavirus used to make damages in the pig industry (52). The number of documented human isolates varied in years, and there are three peaks that reflect the outbreaks of SARS-CoV in 2003, MERS-CoV in 2014–2015 and SARS-CoV-2 in 2019–2020 separately. Using all documented coronavirus genomes in CoVdb, we generated a phylogenetic tree (Figure 2A), from which we observed that the nearest nonhuman isolate to 2019-nCoV is Bat_MN996532 (Bat-CoV-RaTG13), isolated from *Rhinolophus affinis*, a species of bat in the Rhinolophidae family. Strains isolated from pangolins are also in the vicinity of SARS-CoV-2. Pangolin was once considered as a potential intermediate host of SARS-CoV-2 (53). We developed search tools to enable users to search in the big phylogenetic tree (Figure 2B).

In average, there are 5–14 possible ORFs or genes in one coronavirus strain. We grouped homologous coronavirus genes (requiring identity >0.5 and coverage >0.8) into 628 clusters (for details, see Materials and Methods). This number indicates that the differentiation or diversity within coronavirus strains is not low. For these, we still performed a subcellular localization analysis for the 628 clusters to predict their roles in infection, although the structure of coronavirus is not complex. Based on prediction only, 21% (133 items) are predicted to be located in the host nucleus or host cytoplasm, while 40% (250 items) are predicted to be membrane proteins (Figure S1). CoVdb has included more than 50 000 function annotations and more than 300 000 GO records. Using WEGO (54), we found coronavirus genes enrich in the membrane (Figure S2). We searched for possible protein 3D structure for coronavirus genes in the Protein Data Bank (28, 29) and found more than 3 000 000 mappings with an *E*-value <0.05 and a coverage >50%.

For all coronavirus strains, using nine representative human coronavirus genomes as the reference, we did sliding window analyses on Pi (55), Tajima’s D (56), composite likelihood ratio (CLR) (57, 58) and Fst (40). For Pi, Tajima’s D and CLR, the target group are strains that belong to COVID-19, MERS, SARS or other human coronavirus diseases. We also did the same thing for human isolates, bat isolates and isolates of other hosts documented in CoVdb. Fst is between human coronavirus and one nonhuman coronavirus. All these data can be viewed in the genome browser.

### Interface and analysis tools

The genome browser (GBrowser) in CoVdb follows a style with gene segments followed by analysis tracks (CLR, Pi, Tajima’s D and Fst). Users can view population genetic tracks of strains belonging to one or more hosts, such as avian, bat and so on. They can also view that of strains belonging to one or more specific diseases, such as COVID-19, SARS and 229E. Users can select by checkboxes. At the top of GBrowser, there are browsing tools, such as search by inputting a chromosome position, zoom in/out and position movements. It provides notes for whether a gene of ORF is already annotated in GeneBank or newly predicted in the top of the genome browser page. In addition to basic information, CoVdb shows a gene in function, subcellular localization, topology and protein structure. The search engine in CoVdb is powerful and supports fuzzy search, search with taxonomy (such as Alpha, Beta, Sarbecovirus, etc.), filtering, sorting, BLAT and BLAST. CoVdb also allows to search by cell location. For personalized analyses, CoVdb is able to provide gene links if inputting a list of chromosome positions or gene accessions.

**Figure 3.**
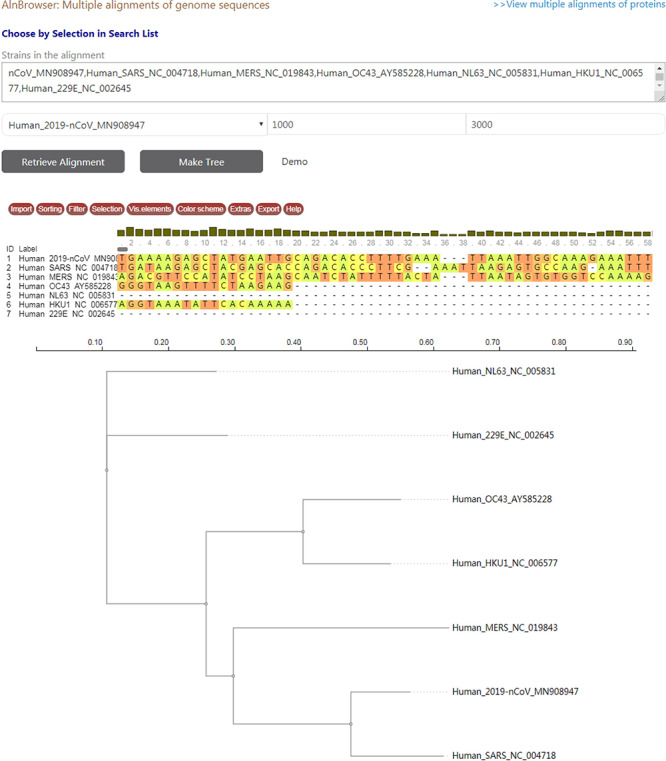
A snapshot displaying the usage of ‘Aln Browser’, where users need to select the reference strain, the start position, the end position and the strains to be put in alignment. If clicking on the button ‘Retrieve Alignment’, a multiple alignment of selected strains will be shown below. If clicking on ‘Make Tree’, a phylogenetic tree will be built basing on the alignment and shown at the bottom.

CoVdb has tools to facilitate some specific use in coronavirus research, such as tracing origination, vaccine or drug design. In the tool ‘Protein’, the protein structure information is listed, where users can view the overlapped amino acids of a coronavirus protein in the 3D structure counterpart and do online protein structure analysis by an embedded application iCn3D (49) (Figure S3A, C). Users also can BLAST a protein sequence against CoVdb’s protein data and view the mapped region in a protein 3D view (Figure S3B, D). The tool ‘Aln Browser’, alignment browser, allows users to retrieve the multiple alignment of two or more strains at some position and build a phylogenetic tree using the alignment (Figure 3). With the tool ‘Pop Analyzer’, population genetics analyzer, users can do personalized online sliding window analyses. Users can choose the window size, the step size, the target region and one or more population genetic tests (Figure S4). Users can jump to the search list and select multiple strains to do analysis in ‘Aln Browser’ or in ‘Pop Analyzer’. For initial users, they can learn to use each analysis tool by clicking the button ‘Demo’. ‘Phylo Tree’ is a tool to view and search in phylogenetic trees made by genomic or proteomic sequences (Figure 2B). Users also can go to the GBrowser page by clicking the name of one strain.

### View population genetic tracks of the spike glycoprotein in GBrowser

The spike glycoprotein (S protein) plays a key role in the infection of COVID-19 while the receptor-binding domain (RBD) (59) is the region in S protein to interact with the human protein ACE2 (60). In CoVdb’s GBrowser (Figure S5), we observed that S protein is highly conserved within SARS-CoV-2/2019-nCoV strains (CLR, Pi and Tajima’s D are nearly fixed to zero for most points). In comparison, for SARS-CoV, there are CLR peaks and variations in Pi and Tajima’s D. For human coronavirus, the region near RBD is highly conserved (with a lowland of Pi and Tajima’s D, while Tajima’s D is −0.81 in median) compared to the region far away from RBD (with a plateau of Pi and Tajima’s D, while Tajima’ D is 1.39 in median). However, we did not observe a similar pattern for bat coronavirus. Human and bat coronavirus are of CLR peaks at different sites. We also observed Fst peaks between human and bat coronavirus. These indicated that the evolution is different not only between SARS-CoV-2/2019-nCoV and SARS-CoV but also between human and bat coronavirus.

## Conclusion

Dedicated to assist researchers to combat the pandemic of COVID-19 and to provide a more specialized platform for coronavirus, we comprehensively gathered data and systematically constructed the coronavirus database, CoVdb. In the database, researchers can conveniently retrieve genomic or gene information of coronavirus and do online analyses in comparative genomics, protein structure and evolutionary biology. With the help of this database, we have successfully developed test strips able to detect SARS-CoV-2 (unpublished). With the increase of the number of sequenced coronavirus genomes, we will provide continuous update and maintenance of the database in the future. Hopefully, this database will play more important roles in fighting against the infection of coronavirus in the future.

## Data Availability

All CoVdb data are publicly and freely accessible at http://covdb.popgenetics.net. Feedback on any aspect of the CoVdb and discussions of coronavirus gene annotations are welcome by email to zhuzl@cqu.edu.cn or mg@cau.edu.cn.
