# Supplementary material for: A database resource and online analysis tools for coronaviruses on a historical and global scale
Source: Database (Oxford). 2021 Aug 5;2020:baaa070. doi: 10.1093/database/baaa070 (PMC7665380; doi:10.1093/database/baaa070)
Supplement: baaa070_Supp [file baaa070_supp.zip › Supplementary Figures.pdf]

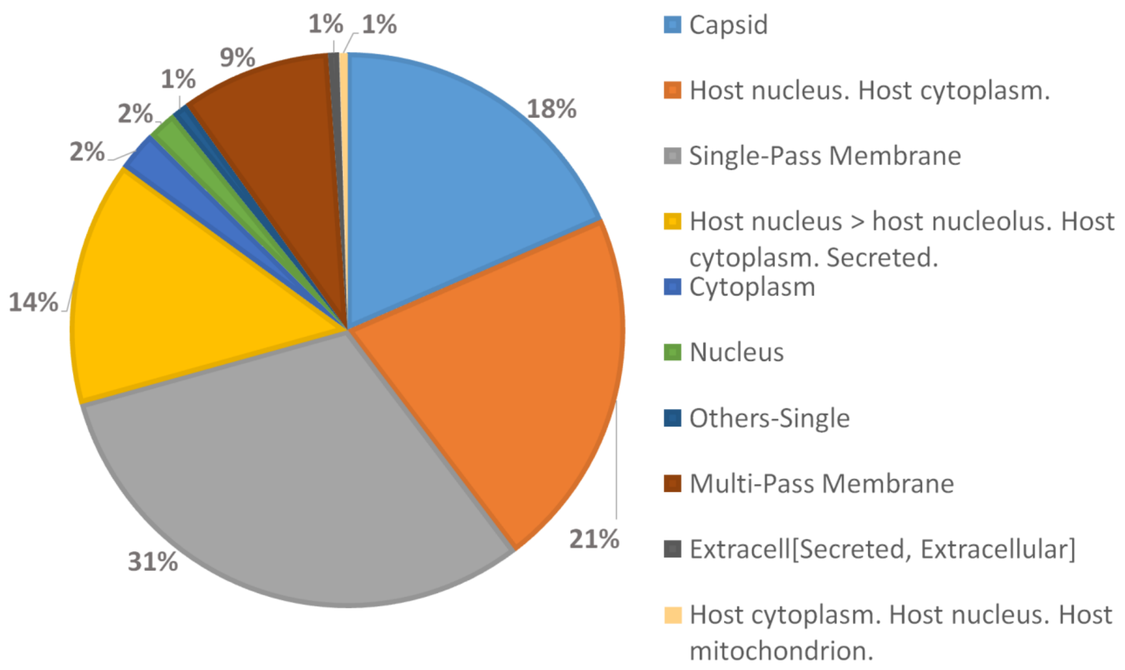

Figure S1. The percentages of conoravirus gene clusters in subcellular locations.

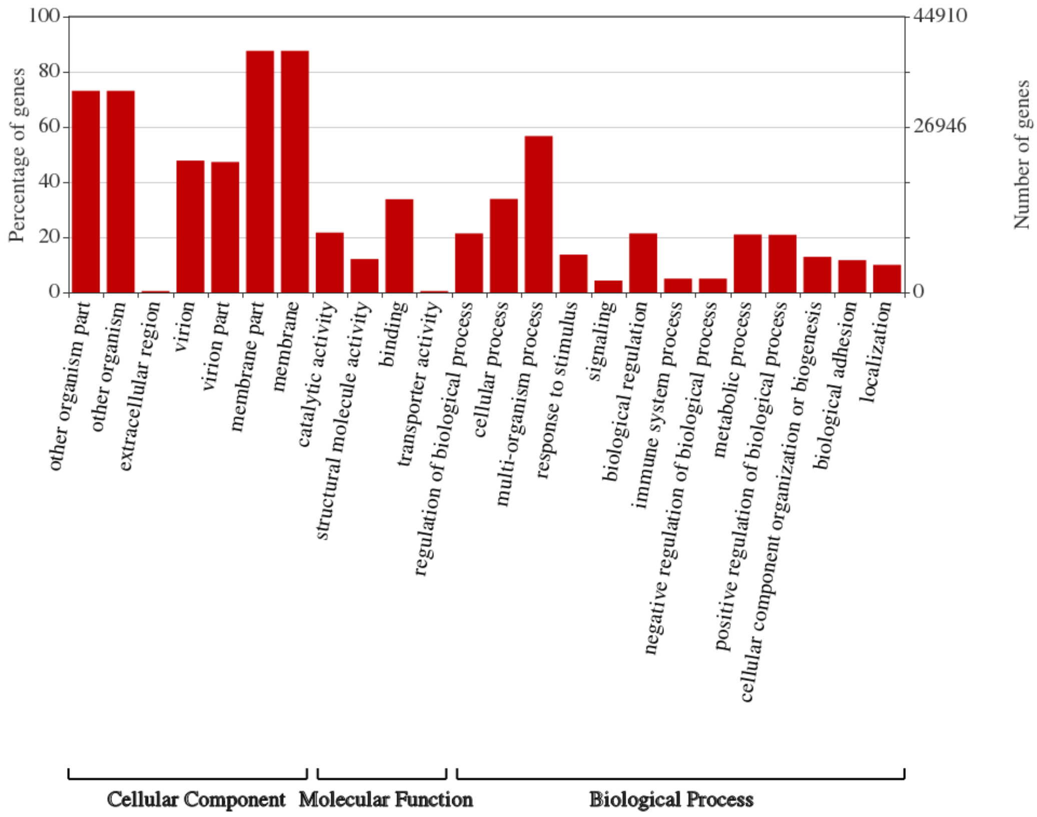

Figure S2. Gene ontology (GO) enrichments of conoravirus genes. This figure was drawn by WEGO.

A

Strain: Human_2019-nCoV_MN908947 Gene: QHD43418.1 E **envelope protein**

Pfam: NS3_envE

PDBID	score	Evalue	Identity	Align-Len	Cov. (Q)	Cov. (T)	Q-Span	T-Span	Analyze in iCn3D
5X29	58.2	4e-13	88.71	62	82.67%	76.54%	4 - 65	20 - 81	Analyze View alignments
2MM4	56.2	2e-12	91.38	58	77.33%	100.00%	8 - 65	1 - 58	Analyze View alignments

B

« BACK

Results from BLAST in the gene model protein sequences : 1 hits

Query	Subject	Identity	Alignment Len.	Mismatches	Gaps	Q.start	Q.end	S.start	S.end	Evalue	Bit Score	3D
Your_seq	QHD43418.1	100.000	24	0	0	1	24	23	46	3.46e-13	47.8	View

C

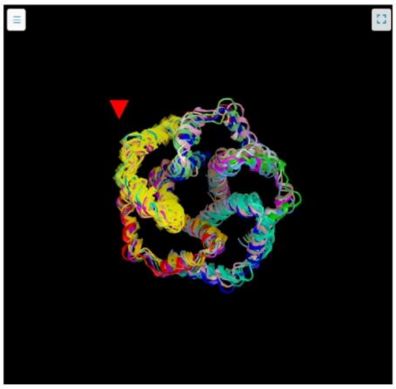

D

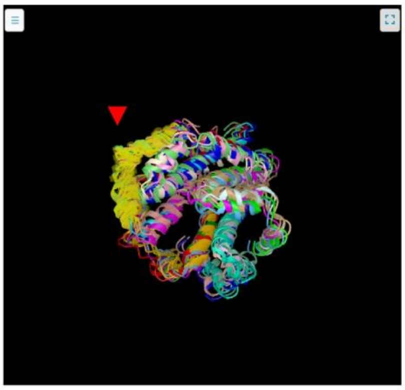

Figure S3. A introduction of the tool "Protein". A is a part of listed protein 3D structure information of genes in the strain Human_2019-nCoV_MN908947. B is a snapshot of the result after online BLAST by querying "FVVFLLVTLAILTALRLCAYCCNI" in proteins of the strain Human_2019-nCoV_MN908947. C is a snapshot of the protein 3D view after clicking the button "Analysis" in A. Here, a red arrow points to the overlapped region of the Coronavirus gene QHD43418.1 and its 3D structural counterpart 5X29. C is a snapshot of the protein 3D view after clicking the button "View" in B. Here, a red arrow points to the overlapped region of the query sequence and the target protein structure 5X29.

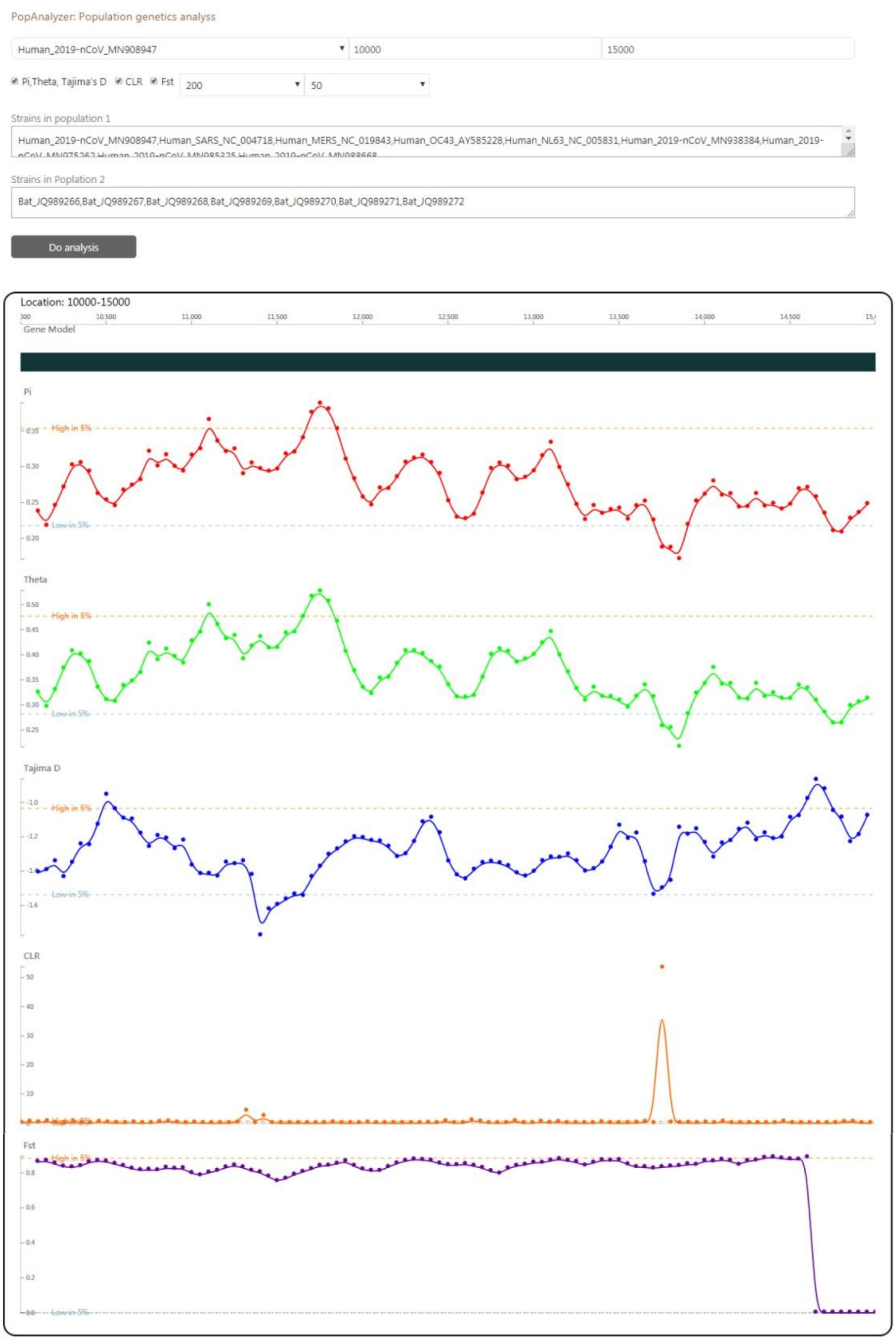

Figure S4. A snapshot of the tool “Pop Analyzer”.

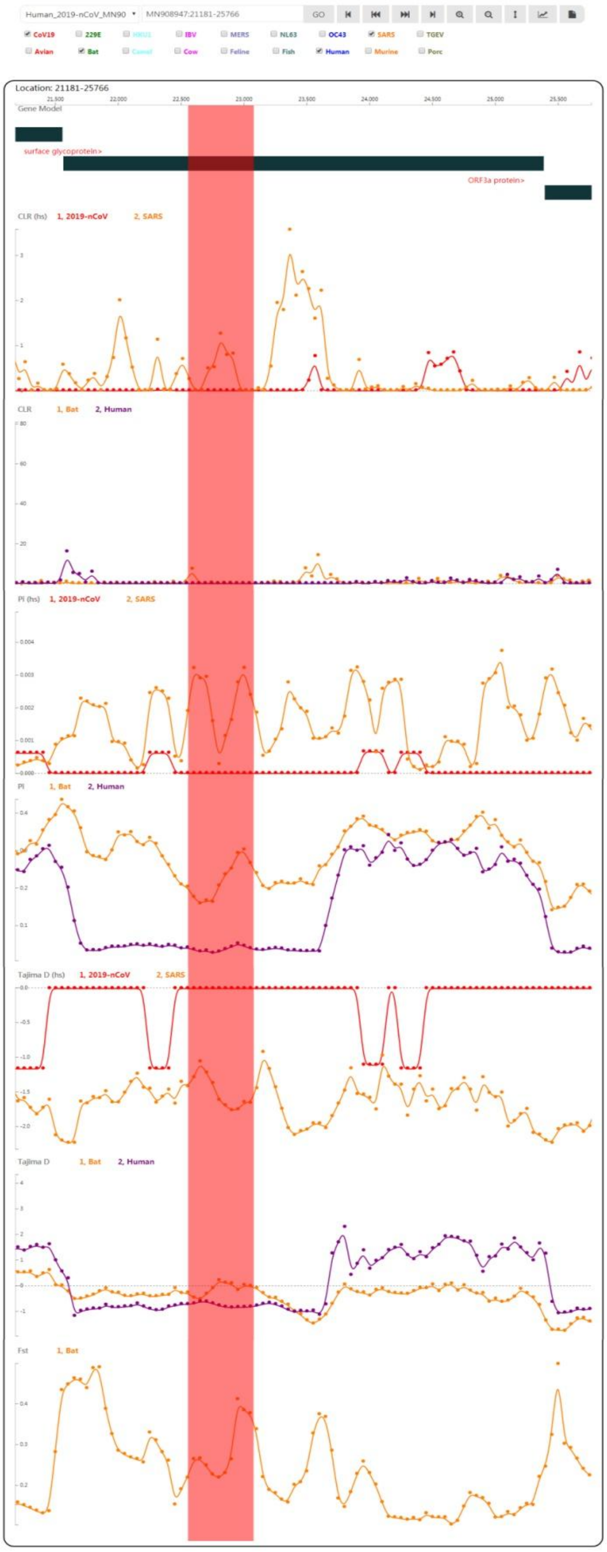

Figure S5. A snapshot of Human_2019-nCoV_ MN908947's spike glycoprotein in GBrowser with RBD (331AA to 550AA in the spike glycoprotein) marked by a red bar. Tracks of population genetic tests are listed following gene segments. "CLR (hs)" are CLR values within 2019-nCoV (SARS-CoV-2) strains (181 samples) and within SARS-nCoV strains (302 samples). "CLR" are CLR values within human isolates (972 samples) and bat isolates (176 samples). So do Pi and Tajima's D. Fst is calculated between human isolates and bat isolates.
